# Sexual function and activity in young adults with a primary brain tumor—A population-based longitudinal study

**DOI:** 10.1093/nop/npag048

**Published:** 2026-05-22

**Authors:** Charlotta Bergström, Lars E Eriksson, Christel Hedman, Roger Henriksson, Claudia Lampic, Lena Wettergren

**Affiliations:** Department of Women’s and Children’s Health, Karolinska Institutet, Stockholm, Sweden; Department of Surgery and Urology, Danderyd Hospital, Stockholm, Sweden; Department of Neurobiology, Care Sciences and Society, Karolinska Institutet, Huddinge, Sweden; School of Health and Medical Sciences, City St George’s, University of London, London, UK; Department of Molecular Medicine and Surgery, Karolinska Institutet, Stockholm, Sweden; R&D Department, Stockholms Sjukhem Foundation, Stockholm, Sweden; Institute for Palliative Care, Region Skåne, Lund University, Lund, Sweden; Department of Clinical Sciences Lund, Lund University, Lund, Sweden; Department of Radiation Science and Oncology, University Hospital, Umeå, Sweden; Department of Public Health and Caring Sciences, Uppsala University, Uppsala, Sweden; Department of Psychology, Umeå University, Umeå, Sweden; Department of Women’s and Children’s Health, Karolinska Institutet, Stockholm, Sweden; Department of Public Health and Caring Sciences, Uppsala University, Uppsala, Sweden

## Abstract

**Background:**

There is limited knowledge about how diagnosis and treatment for brain tumors affect intimacy and sex life in young adults. This study examined sexual function and activity and identified factors associated with sexual function in a national cohort of young adults up to 5 years after being diagnosed with a primary brain tumor.

**Methods:**

Patients diagnosed with a malignant or benign brain tumor at ages 18-39 years were identified through the Swedish Quality Registry for CNS tumors and approached with a comprehensive survey at 1.5-, 3-, and 5-year postdiagnosis. In total, 123 responded (58%) and 72 (31 men and 41 women) of them completed the full set of surveys. Sexual function was assessed with the PROMIS SexFS v2.0. Changes over time were examined using repeated measures ANOVA, and multivariable linear regression models were conducted to identify factors associated with sexual dysfunction.

**Results:**

Most participants were sexually active (>80%) and satisfied with their sex life, although a substantial proportion reported low interest in sexual activities. No changes over time were observed in the domains Satisfaction with sex life (*F*(2, 104) = 0.49, *P* = .66) or Interest in sexual activity (*F*(2, 136) = 0.58, *P* = .56). Sexual dysfunction was associated with depressive symptoms and body image disturbance. Clinical characteristics were not associated with sexual dysfunction.

**Conclusions:**

Most young adults diagnosed with a brain tumor were sexually active and reported satisfaction with their sex life. A subgroup reported low interest in sexual activities, underscoring the need to include discussions about possible cancer-related impact on sex life into follow-up care.

Key Points

- Most participants were sexually active up to 5 years after diagnosis.
- Overall, few participants reported sexual dysfunction.
- Sexual dysfunction was associated with psychological factors.

Importance of the Study

Evidence on sexual function among young adults with primary brain tumors is scarce, typically limited by cross-sectional studies and retrospective assessments. Consequently, the impact of brain tumors and their treatments on sexual health remains poorly understood, contributing to limited clinical guidance. This population-based longitudinal study follows a national cohort of individuals diagnosed with a primary brain tumor in young adulthood (18-39 years). Participants were assessed at 3 time points (1.5-, 3-, and 5-year postdiagnosis) using standardized instruments. Among the 72 individuals completing all surveys, the majority were sexually active and reported overall satisfaction with their sex life. Most concerns were related to low interest in sexual activities. Sexual dysfunction was associated with self-reported symptoms of depression and body image disturbance. These findings underscore the importance of including discussions about sex life into follow-up care, in order to identify individuals who may need support.

In Sweden, approximately 1400 individuals are diagnosed with a primary brain tumor annually,^1^ of which about 10% are aged 18-39 years,^2^ commonly defined as young adults.^3^ This group has a distinct tumor distribution and histological profile compared to other age groups.^4^ Brain tumors in young adults comprise both benign tumors, with meningiomas being the most common, and malignant tumors, with gliomas being the most prevalent.^4^ Management of meningiomas typically includes surveillance, surgery, and in some cases radiotherapy, primarily aimed at controlling tumor growth and preventing recurrence.^5^ Prognosis for meningiomas is generally excellent, with 5-year survival exceeding 95% in young adults.^6^ In contrast, treatment for gliomas is generally more intensive involving a combination of surgery, radiotherapy, and/or chemotherapy. The specific regimen depends on tumor type and grade,^7^ and survival varies markedly by subtype. Among individuals aged 20-39 years, 5-year survival is close to 70% for all malignant CNS tumors combined, but falls to 27% for those diagnosed with glioblastoma.^8^ Swedish registry data indicate a 5-year survival rate of approximately 40% for high-grade glioma in this age group, reflecting the prognostic advantage associated with younger age despite the aggressive nature of the disease.^9^ The impact on health-related quality of life can be substantial for individuals with both malignant and benign tumors.^10–12^ Physical and psychosocial sequalae, including memory and attention deficits, motor impairments, fatigue, sleeping disorders, anxiety and depression, are commonly reported.^12^ A recent population-based study with long-term follow-up of young adults with brain tumors, revealed a high burden of persistent health challenges, particularly fatigue, neurocognitive difficulties and uncertainty about the future, which were significantly more prevalent than among those with other cancer types.^11^

A brain tumor diagnosis during young adulthood presents unique challenges, as this time in life includes critical milestones such as completing education, establishing a career, forming romantic relationships and building a family.^13^ Sexuality is an integral part of life^14^ and young adults with cancer consistently report unmet needs in this area.^15^ However, previous research on cancer-related impact on sex life among young adults with cancer has predominantly focused on individuals with reproductive cancers, such as breast^16^ and testicular cancer.^17^ Consequently, sexual function in young adults diagnosed with other malignancies, including primary brain tumors, has received little attention. A population-based study showed that patients with primary brain tumors received significantly less information about the potential impact of treatment on their sex life compared to patients diagnosed with other cancers.^18^ One possible explanation could be health-care professionals’ limited knowledge of how sexual functioning may be affected in this group of patients.

Sexual problems in patients with reproductive cancers have been associated with female gender,^19^ treatment intensity,^16^^,^^19^^,^^20^ psychological distress^20^ including body image disturbance.^16^ Concerns with body image (eg weigh gain/loss, scars), may influence self-esteem, which in turn can affect desire, intimacy, and sexual activity. Similarly, emotional distress may negatively impact on self-esteem and well-being, contributing to lower interest in sexual activities.

Of the few studies that have investigated potential impact on sex life in individuals with brain tumors,^21–25^ only 4 have included a substantial proportion of young adults. Surbeck et al^21^ studied 32 individuals who had returned to work after surgery for diffuse low-grade glioma and found that over half of the women and a third of the men reported sexual dysfunction. In a study of 46 hospitalized or day-care patients with various types of brain tumors, more than half experienced sexual problems, most commonly lack of desire.^22^ These problems were associated with poorer quality of life, anxiety, and depression. A cross-sectional study of 14 individuals with low-grade gliomas (response rate 21%) found that their scores on the EORTC QLQ-SH22 sexual satisfaction scale were comparable to reference values from cancer patients with no evidence of disease.^25^^,^^26^ Lastly, a recent study of 148 patients with low- and high-grade glioma found that a substantial proportion experienced a negative impact on their sexual well-being, including reduced engagement in partner sex, disrupted relationships, and diminished feelings of sexual attractiveness.^24^ However, erectile function and vaginal lubrication were largely unaffected.^24^

In summary, although 4 previous studies have investigated the impact of a brain tumor on sex life, these studies were limited by small and selected samples, retrospective or cross-sectional designs. Therefore, it remains unclear how common sexual dysfunction is in this population, which aspects of sexual functioning are most affected, and how these problems evolve over time. Greater understanding of the prevalence and characteristics of sexual dysfunction is essential to improve care of young adults with brain tumors. The present study therefore offers a novel contribution by providing a population-based sample and a longitudinal design using standardized measures up to 5 years postdiagnosis. Additionally, clinical data were retrieved from the Swedish Quality Registry for CNS tumors. This study aimed to examine sexual function and activity, and to identify factors associated with sexual function in a national cohort of young adults up to 5 years after being diagnosed with a primary brain tumor.

## Materials and Methods

### Study Setting and Design

This prospective study is part of the Swedish population-based Fertility and Sexuality following Cancer (Fex-Can) Cohort study,^27^ which maps sexual and reproductive health in young adults diagnosed with selected cancers up to 5 years postdiagnosis. The present study focuses on sexual function and satisfaction among men and women with brain tumors by combining survey data with clinical data retrieved from the National Quality Registry for Brain Tumors.^9^^,^^28^^,^^29^ The study is outlined in accordance with the STROBE guidelines.^30^ Ethical approval was obtained from the Regional Ethical Review Board in Stockholm (Record No: 2013/1746-31/4; 2014/2244-32; 2017/916-32; 2017/1416-32).

### Participants and Procedure

**Figure 1.**
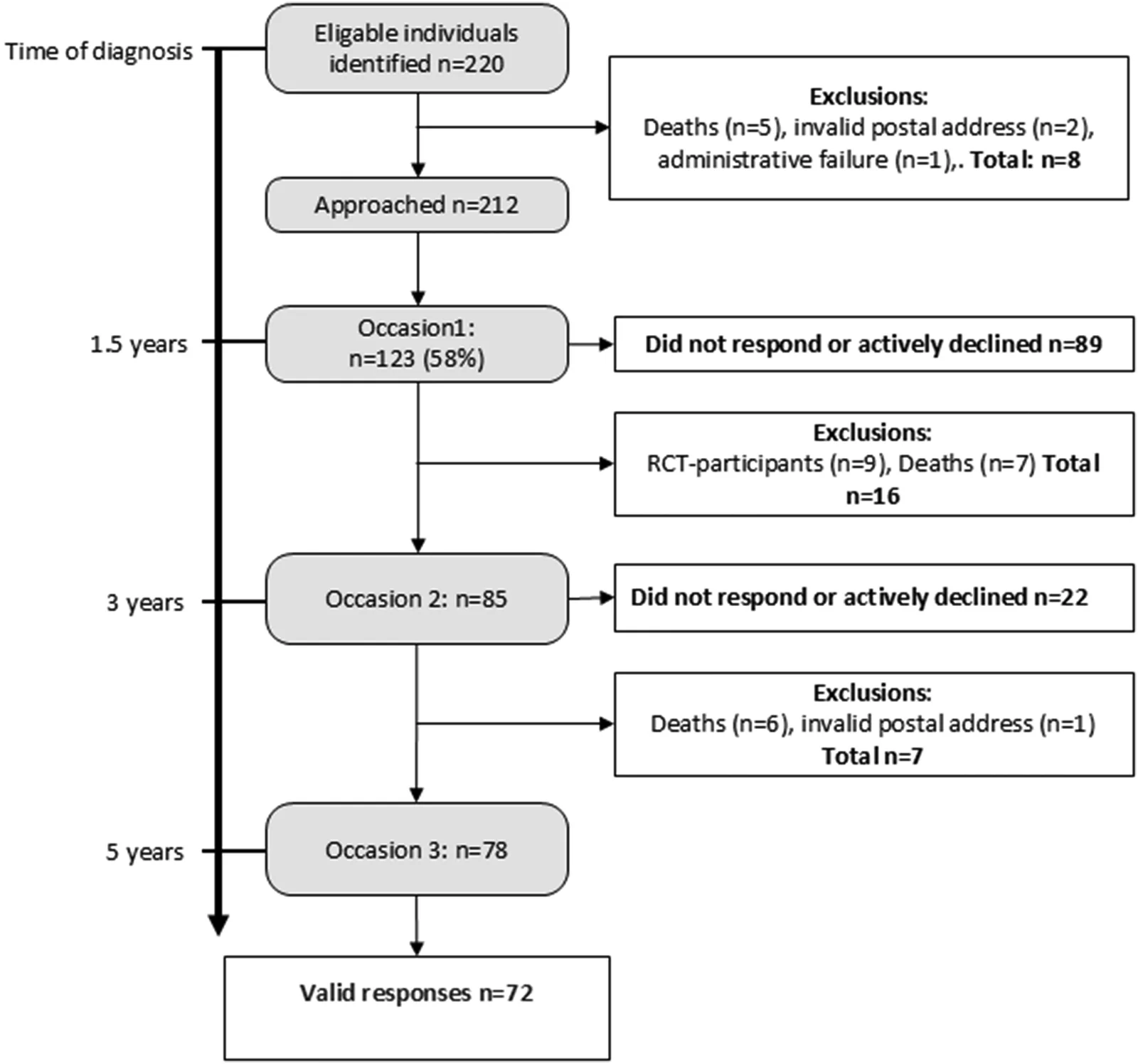
Flowchart of the study participants.

All individuals in Sweden diagnosed with a primary brain tumor at the age of 18-39 years, from January 2016 through August 2017, were identified through the National Quality Registry for CNS tumors, see study protocol.^27^ Eligible patients were invited to complete a comprehensive survey at 1.5 years postdiagnosis. Of 212 individuals, 123 accepted study participation (response rate 58%) and 72 participants completed the comprehensive survey at all time points: 1.5-, 3-, and 5-year postdiagnosis, see Figure 1. Informed consent was obtained from all participants.

### Measures

From the comprehensive survey, we selected variables measuring sexual function and activity, body image, symptoms of anxiety and depression, and sociodemographics. Clinical data (diagnosis, treatment) were retrieved from National Quality Registry.

Sociodemographic variables were collected from the first assessment and included birth country, education, occupation, relationship status, children and sexual orientation.

Sexual function and satisfaction were assessed with the Patient-Reported Outcomes Measurement Information System (PROMIS) Sexual Function and Satisfaction Measure (SexFS) version 2.0.^31^ For the present study 4 generic domains were selected: Satisfaction with sex life (2 items), Interest in sexual activity (2 items), Orgasm—pleasure (1 item), Orgasm—Ability (1 item). Additionally, 2 body-part specific domains were used: Erectile function (3 items) and Vaginal lubrication (2 items). Participants responded based on their experiences during the past month, using a 5-point Likert scale. Additionally, questions regarding sexual activity with and without a partner during the past month were included with sexual activity defined as sex with a partner and/or solo sex (masturbation). The items of the domain Interest in sexual activity are completed by all individuals, whereas for the remaining domains, items are only completed by those who have been sexually active the last 30 days. Domain scores were transformed to a *t*-score metric, where 50 represents the mean for the American general population (standard deviation [SD] = 10).^31^ One SD below or above 50 (depending on the direction of the scale) in the respective domain is considered as indicative of dysfunction.^31^ Additionally, 1 item assessing perceived bother with one’s level of sexual interest was included: “How much has the level of your interest in sexual activity bothered you?” Bother related to sexual interest was rated on a 5-point Likert scale and dichotomized (none/little vs some/quite a lot/very much). The SexFS has shown adequate construct, content, known-groups validity and test-retest reliability.^31^^,^^32^ The Swedish version has shown satisfactory psychometric properties.^33^

Symptoms of anxiety and/or depression were assessed using the Hospital Anxiety and Depression Scale (HADS).^34^ The HADS includes 2 subscales comprising 7 items each, scored from 0 to 3. Each subscale yields a total score ranging from 0 to 21, with higher scores indicating more anxiety or depression symptoms. The HADS has shown satisfactory validity and internal consistency.^35^

Body image disturbance was assessed using the Body Image Scale (BIS) developed to assess body image in patients with cancer.^36^ The BIS consists of 10 items rated on a 4-point scale (0-3). Total scores range from 0 to 30, with higher scores indicating greater body image disturbance. The scale has demonstrated satisfactory reliability and clinical validity.^36^

Clinical data was obtained from the Swedish Quality Registry for CNS tumors,^9^^,^^29^ and included gender, cancer type and stage, date of diagnosis, and received primary treatment. Based on received treatment, each patients’ treatment intensity was classified into 4 levels (least, moderately, very or most intensive/extensive treatment) according to the Intensity of Treatment Rating-Young Adult scale, the ITR-YA.^37^ The ITR-YA was developed through iterative expert review rounds within the Fex-Can project. The evaluations demonstrated high interrater reliability and clear known-groups validity, confirming that the scale provides a consistent and clinically meaningful measure of treatment intensity.^37^

### Statistical Analysis

Analyses of dropouts and attrition were conducted by comparing sociodemographic and clinical characteristics between study participants (*n* = 72), initial nonresponders (*n* = 89), and those who completed only 1 or 2 of the additional surveys (*n* = 42). Categorical variables were analyzed using chi-square tests or Fisher’s exact test, and continuous variables using independent *t*-tests.

Sexual function and satisfaction, sexual activity and bother related to interest in sexual activity, are presented descriptively, with numbers and percentages of individuals reporting sexual function at each assessment by gender. Sexual dysfunction is defined as, 1 SD below or above the norm population (depending on the direction of the scale) in at least 1 domain.^31^

Crosstabulation analyses were conducted to examine the association between low interest in sexual activity and self-reported bother related to one’s level of interest in sexual activity. Odds ratios (ORs) were calculated to estimate the odds of being classified as having dysfunction among participants reporting bother compared to those not reporting bother, stratified by sex and on all occasions.

A repeated measures ANOVA^38^ was conducted to examine change across time points for 2 of the domains: Interest in sexual activity and Satisfaction with sex life. Time (3 levels) was treated as a within-subjects factor. Mauchly’s test of sphericity indicated that the assumption of sphericity was not violated for the domains Interest in sexual activity (*W* = 0.935, χ²(2) = 4.48, *P* = .106) or for Satisfaction with sex life (*W* = 0.988, χ²(2) = 0.60, *P* = .741); therefore, no Greenhouse-Geisser correction was applied. Effect sizes were reported as *ω*² and interpreted as: *ω*² < 0.01 = trivial, 0.01 = small, 0.06 = medium, and 0.14 = large.^39^ To identify factors associated with sexual dysfunction, multivariable linear regression models were conducted for the same 2 domains at all 3 points. Effects were expressed as unstandardized regression coefficients (*β*). Potential associated factors were selected a priori based on the literature: gender^19^ (man/woman), age at diagnosis^19^^,^^20^ (continuous), level of education^40^ (university degree/no university degree), relationship status^19^^,^^20^^,^^41^ (partnered/not partnered), tumor type (benign/malignant), treatment intensity^16^^,^^19^^,^^20^ (less intensive/more intensive), body image disturbance^16^^,^^17^^,^^20^ (continuous), and symptoms of anxiety^22^ (continuous) and symptoms of depression^22^ (continuous). First, each factor was examined in bivariate analyses. Factors associated with sexual dysfunction in the respective domain were then included in the multivariable models.

Missing data in the SexFS were handled according to the established PROMIS methodology,^31^ and for the BIS and the HADS, the individual’s mean was imputed when single items on respective scales were missing, provided that at least half of the items were answered.

Statistical analyses were conducted with SPSS Statistics for Windows, version 30 (IBM Corp.). All tests were 2-tailed, and *P*<.05 were considered statistically significant.

## Results

### Participant Characteristics

The sample comprised 31 men and 41 women, with a mean age at diagnosis of 30 years Overall, 38 participants (55%) had malignant tumors, comprising 19 men (63% of all men) and 19 women (48% of the women). The majority had undergone surgery, both among those with benign (87%) and malignant tumors (94%). In addition, 10% of participants with benign tumors and 31% with malignant tumors had received radiotherapy and/or chemotherapy. More details are shown in Table 1. World Health Organization classification as well as International of Diseases for Oncology, 3rd Edition (ICD-O-3) codes of the study participants’ tumors are presented in Table S1. Most participants were partnered at 1.5 years after diagnosis (men 74%; women 83%), and these proportions remained largely unchanged at the 3- and 5-year assessments (data not shown).

**Table 1.** Sociodemographic and clinical characteristics of young adults with a primary brain tumor 1.5 years after diagnosis (*n* = 72).

	Study cohort *n* (%)	Men *n* = 31 *n* (%)	Women *n* = 41 *n* (%)
Sociodemographic variables			
Age at diagnosis, years; mean (SD)	30.5 (6.15)	29.4 (6.07)	31.3 (6.14)
Country of birth			
Sweden	61 (85)	26 (84)	35 (85)
Outside Sweden	11 (15)	5 (16)	6 (15)
Highest education			
University degree	40 (56)	13 (42)	27 (66)
No university degree^a^	32 (44)	18 (58)	14 (34)
Main occupation			
Working/studying	46 (64)	20 (65)	26 (63)
Unemployed, sick-leave, other^b^	26 (36)	11 (35)	15 (37)
Relationship status			
Partnered	57 (79)	23 (74)	34 (83)
Not partnered	15 (21)	8 (26)	7 (17)
Have children			
Yes	35 (49)	11 (35)	24 (58)
No	37 (51)	20 (65)	17 (42)
Sexual orientation			
Heterosexual	69 (97)	29 (97)	40 (98)
Nonheterosexual	2 (3)	1 (3)	1 (2)
Clinical variables			
Tumor type			
Meningioma	29 (42)	9 (30)	20 (51)
Benign tumors	2 (3)	2 (6)	0 (0)
Low malignant tumors	11 (16)	5 (17)	6 (16)
High malignant tumors	27 (39)	14 (47)	13 (33)
Missing	3	1	2
Primary treatment^c^			
Surgery	63 (89)	27 (90)	36 (80)
Chemotherapy	15 (21)	8 (27)	7 (17)
Radiotherapy	15 (21)	9 (30)	6 (15)
Treatment intensity^d^			
Least/moderate	53 (76)	21 (70)	32 (80)
Very/most	17 (24)	9 (30)	8 (20)
Currently on treatment (self-reported)			
Yes	11 (15)	10 (32)	1 (2)
No	61 (85)	21 (68)	40 (98)
Psychological factors			
Anxiety symptoms (HADS-A), mean (SD)	6.68 (4.24)	5.22 (4.39)	7.78 (3.82)
Depressive symptoms (HADS-D), mean (SD)	4.47 (3.38)	4.03 (3.68)	4.81 (3.32)
Body image disturbance (BIS), mean (SD)	7.03 (5.88)	6.08 (5.43)	7.75 (6.17)

Abbreviations: BIS, body image scale; HADS-A, Hospital Anxiety and Depression Scale-Anxiety; HADS-D, Hospital Anxiety and Depression scale-Depression; SD, standard deviation.

a Includes elementary school, upper secondary school, folk high school.

b Parental leave, retired.

c Sum up to more than 100% as participants may have had more than 1 treatment.

d Treatment intensity according to the Intensity of Treatment Rating-Young Adult (ITR-YA).

Analyses of baseline dropouts revealed no significant differences in clinical characteristics (gender, age at diagnosis, tumor type, primary treatment, or treatment intensity) between responders (*n* = 72) and initial nonresponders (*n* = 89) (Table S2). Attrition analyses showed no significant differences in sociodemographic or clinical characteristics between participants who completed all 3 surveys and those who completed only 1 or 2 of the surveys (Table S2).

### Sexual Activity and Function

At all assessments, a majority of both men and women reported having been sexually active (partner sex and/or masturbation) during the past month. More than 80% of the men and about 75% of the women rated that they were satisfied with their sex life at all time points (Table 2).

**Table 2.** Number and proportions of patients rating sexual activity and function by gender (*n* = 72), at 1.5-5 years postdiagnosis.

	Men (*n* = 31)			Women (*n* = 41)		
	1.5 years *n* (%)	3 years *n* (%)	5 years *n* (%)	1.5 years *n* (%)	3 years *n* (%)	5 years *n* (%)
Sexual activity^a^ the past 30 days						
Sexually active	24 (77)	27 (87)	26 (84)	34 (83)	35 (85)	37 (90)
Sexually activity with a partner	15 (63)	15 (56)	18 (69)	31 (91)	32 (91)	29 (78)
Domains of sexual function^b^						
Satisfaction with sex life	20 (83)	24 (89)	23 (88)	30 (88)	27 (77)	29 (78)
Interest in sexual activity	21 (68)	26 (84)	25 (86)	29 (72)	22 (54)	24 (58)
Orgasm ability	23 (96)	23 (85)	24 (92)	27 (79)	24 (69)	22 (59)
Orgasm pleasure	23 (96)	25 (96)	26 (100)	34 (100)	29 (85)	34 (92)
Erectile function	24 (100)	26 (96)	26 (100)	n/a	n/a	n/a
Vaginal lubrication	n/a	n/a	n/a	32 (94)	29 (83)	31 (84)

Abbreviations: n/a, not applicable.

a Sexual activity is defined as sex with a partner and/or solo sex (masturbation).

b Patient-Reported Outcomes Measurement Information System (PROMIS) Sexual Function and Satisfaction (SexFS), version 2.0. Self-reported function defined as cutoff <1SD above/below the t-score mean of the norm population. Completed by individuals who have had sexual activity (with or without partner) during the past 30 days.

Valid percentages.

The majority of both men and women reported high levels of sexual function on all occasions as presented in Table 2. About two-thirds of both men and women reported being interested in sexual activity at 1.5 years postdiagnosis. Erectile function in men and vaginal lubrication in women remained consistently favorable across all assessments. Nine men (29%) and 11 women (27%) reported bother related to their level of sexual interest at 1.5 years postdiagnosis. The corresponding proportions at 3 years were 13% (*n* = 4) for men and 19% (*n* = 8) for women, and at 5 years, 23% (*n* = 7) and 27% (*n* = 11), respectively. Across all time points, participants scoring in the dysfunction range for Interest in sexual activity consistently had higher odds of reporting bother than those scoring within the functional range. The ORs (dysfunction vs function) at 1.5, 3, and 5 years were 4.25, 1.92, and 5.25 among men, and 1.80, 2.26, and 2.07 among women (data not shown).

### Sexual Function Over Time

A repeated measures ANOVA showed no significant time effect for Interest in sexual activity (*F*(2, 136) = 0.58, *P* = .56, *ω*² < 0.01) or Satisfaction with sex life (*F*(2, 104) = 0.49, *P* = .66, *ω*² < 0.01), respectively (Table 3). Analyses stratified by gender yielded similar results (data not shown).

**Table 3.** Interest in sexual activity and Satisfaction with sex life (PROMIS SexFS) over time in men and women diagnosed with a primary brain tumor in young adulthood.

	1.5 years M (SD)	3 years M (SD)	5 years M (SD)	*P*-value (time)	*ω*²^a^
Interest in sexual activity					
Total *n* = 69	46.65 (11.23)	45.30 (11.50)	45.46 (10.13)	.56	<0.01
Satisfaction with sex life					
Total *n * = 53	51.29 (7.31)	50.13 (7.73)	51.28 (8.22)	.49	<0.01

Mean values represent PROMIS SexFS *t*-scores (possible range 0-100), where higher scores indicate greater interest in sexual activity and higher satisfaction with sex life. Scores are standardized to a reference population (mean = 50, SD = 10).

Abbreviations: M, mean; PROMIS, Patient Reported Outcomes Measurement Information System; SD, standard deviation; SexFS, Sexual Function and Satisfaction measure, version 2.0.

a *ω*² Omega squared (effect size).

### Factors Associated With Sexual Function

Depressive symptoms were negatively associated with Interest in sexual activity (1.5 years) and Satisfaction with sex life (1.5 and 3 years). Similarly, body image disturbance was negatively associated with Interest in sexual activity (3 and 5 years) and Satisfaction with sex life (5 years). Having a partner was associated with greater Satisfaction with sex life (3 and 5 years). Clinical factors were not statistically significantly associated with sexual function at any timepoint (Table 4).

**Table 4.** Results of multivariable linear regression models for sexual function in men and women diagnosed with a primary brain tumor, 1.5-5 years postdiagnosis.

Factors	1.5 years			3 years			5 years		
	*β*	95% CI	*P*-value	*β*	95% CI	*P*-value	*β*	95% CI	*P*-value
Interest in sexual activity									
Gender									
Women vs men (ref)							−2.95	−7.55 to 1.65	.21
Body image disturbance (BIS)				−0.63	−1.07 to −.20	**.005**	−0.40	−0.77 to −0.03	**.03**
Anxiety symptoms (HADS-A)							0.021	−0.64 to 0.68	.95
Depressive symptoms (HADS-D)	−0.79	−1.53 to −0.05	**.04**	−0.60	−1.15 to 0.25	.087	−0.74	−1.49 to 0.02	.054
Satisfaction with sex life									
Relationship status									
Partner vs no partner (ref)				6.81	2.93 to 10.70	**<.001**	7.03	2.25 to 11.83	**.005**
Body image distutrbance (BIS)				−0.32	−0.65 to 0.01	.057	−0.36	−0.69 to −0.04	**.03**
Anxiety symptoms (HADS-A)				−0.07	−0.55 to 0.41	.78			
Depressive symptoms (HADS-D)	−0.73	−1.28 to −0.18	**.01**	−0.73	−1.34 to −0.13	**.02**	−0.50	−1.13 to 0.12	.11

The models included only the factors that were associated with sexual function in the specific domain in bivariate analysis.

Abbreviations: BIS, Body Image Scale; CI, confidence interval; HADS-A, Hospital Anxiety and Depression Scale-Anxiety; HADS-D, Hospital Anxiety and Depression Scale-Depression; PROMIS, Patient Reported Outcomes Measurement Information System; SexFS, Sexual Function and Satisfaction measure, version 2.0.

Statistically significant (*P* < .05) factors in the multivariable models indicated in bold.

## Discussion

To our knowledge, this is the first population-based longitudinal study to examine sexual function and activity in individuals diagnosed with a primary brain tumor. In this cohort of young adults, most were sexually active (with partner sex and/or masturbation) up to 5 years following diagnosis, and the majority reported overall satisfaction with their sex life. Nevertheless, a substantial proportion reported low interest in engaging in sexual activities. Sexual function was associated with psychological factors. Patients rating low interest in sexual activity and satisfaction with sex life were more likely to report higher levels of depressive symptoms and body image concerns at various time points during follow-up. In contrast, clinical characteristics, including tumor type and treatment intensity, were not associated with sexual function.

Our findings indicate less sexual problems following a brain tumor diagnosis compared to previous reports. With the exception of the study of Willmann et al^25^ which assessed 14 individuals with low-grade glioma treated with proton therapy and reported overall satisfaction with sex life, prior studies generally describe a higher prevalence of sexual problems. Surbeck et al,^21^ using a validated measure, reported sexual dysfunction in nearly half of their participants. Other studies relied on retrospective self-reports and found that sexual problems were reported by 25% of patients before diagnosis and 58% after diagnosis.^22^ Further, Leonetti et al^24^ reported that 25% of their patients experienced a deterioration in sexual well-being, and 48% reported reduced engagement in partner sex following diagnosis. Comparisons across the existing studies are limited by small sample sizes and low response rates, such as 21% in Willman et al^25^ and 53% in Surbeck et al.^21^ These methodological limitations likely contribute to differences in reports and may help explain the lower prevalence of sexual dysfunction observed in our cohort. Unlike previous single-center studies with selected samples, our population-based study included both malignant and benign tumors; however, no clinical characteristics were associated with sexual dysfunction. Participants in the present study were relatively young (mean age 31 years). Most women were likely premenopausal, and men were at lower age-related risk for erectile dysfunction. They were followed longitudinally for up to 5 years postdiagnosis, suggesting relatively good health status and favorable prognosis. In contrast, earlier studies included older patients and varied widely in timing of assessment, including participants undergoing treatment.^22^ Moreover, we used the validated PROMIS SexFS with repeated assessments, while prior studies often relied on retrospective, nonvalidated measures,^22^^,^^24^ which are prone to recall bias. Notably, 2 of the retrospective studies^22^^,^^24^ also assessed current sexual problems at the time of the data collection, and Leonetti et al additionally examined erectile function and vaginal lubrication,^24^ which consistent with our findings, appeared largely preserved.

Consistently with Leonetti et al^24^ we found no association between sexual function and tumor type (benign vs malignant). Benign tumors, particularly those situated in critical areas such as the skull base, may require extensive surgical intervention, or adjuvant therapy similar to that of malignant tumors.^42^ These procedures may lead to complications such as facial nerve palsy, manifested by difficulty closing the eye, swallowing dysfunction, or diminished ability to smile.^43^ Such functional changes may have psychosocial impact, which may in turn influence sexual function and intimacy. Furthermore, emotional distress, regardless of tumor type, can contribute to sexual dysfunction. A population-based study has shown that patients with meningioma use antidepressants to a higher degree than people in the general population at 2 years after surgery.^44^ Finally, it is possible that a larger study sample would have shown significant differences with regard to type of tumor.

Our findings indicate that young adults with brain tumors do not have more sexual problems than peers without a history of cancer.^45^ The Fex-Can research project^27^ includes a comparison group of individuals of similar age without a history of cancer, drawn from the Swedish general population (*n* = 819), who completed the same survey.^45^ Self-reported satisfaction with sex life, as well as the proportion of men reporting erectile function and women reporting functional vaginal lubrication, were comparable to those observed in our comparison group. Among men with a brain tumor, interest in sexual activity appeared to be slightly lower 1.5 years after diagnosis compared to men without cancer, but numerically increased over time (not significant), and eventually reached levels comparable to those of men without cancer.^45^ Women with a brain tumor initially reported levels of sexual interest similar to that of women without cancer; however, over time, a slight, nonsignificant decline in sexual interest was observed.

To further contextualize the findings on interest in sexual activity, we analyzed an item assessing bother related to one’s level of sexual interest. This item does however not specify whether one is bothered by high or low interest in sexual activities. Given that most individuals who reported bother also scored low on the domain Interest in sexual activity, it appears that the concerns in most cases were linked to low interest rather than high interest. Future studies should employ qualitative methods to explore how young adults with brain tumors experience impact on sexual desire, sexual functioning, and the relational aspects of their sex life, and how these changes influence their everyday lives. Such insights may reveal unmet needs that are not captured by standardized measures and generate knowledge that can improve communication and psychosocial support in follow-up care.

The association observed between depressive symptoms and sexual dysfunction aligns with previous research showing that depression is linked to reduced sexual desire and impaired arousal.^22^ This reflects core features of depressive symptoms such as diminished motivation, reduced emotional energy, and lowered capacity for pleasure, all of which may limit the ability to initiate or enjoy sexual activity. Moreover, prior studies have shown that concurrent emotional distress and body image disturbance are associated with sexual dysfunction.^20^ For young adults living with a brain tumor, sexual concerns may be further complicated by illness-related anxiety,^11^ fear of recurrence,^11^ cognitive difficulties,^11^^,^^12^ and changes in daily functioning.^12^ Furthermore, low interest in sexual activity was the most frequently reported domain of sexual dysfunction among both men and women, a problem often influenced by emotional distress. Nevertheless, the temporal relationship between emotional distress and sexual dysfunction remains unclear. Depressive symptoms may contribute to decreased interest in having sex, while low sexual interest may in turn exacerbate symptoms of depression.

Although body image concerns may occur after a brain tumor diagnosis, their association with sexual health outcomes remains largely unexplored. Rowe et al^46^ examined body image concerns in 100 adults with various primary brain tumors in mean 5 years postdiagnosis. Approximately one-third reported body image concerns, which were associated with younger age and higher body mass index. Participants described concerns with weight gain, hair loss, and scars. A novel contribution of the present study is the observed association between body image disturbance and sexual function. Concerns with body image (eg weigh gain/loss, scars), may influence self-esteem, which in turn can affect desire, intimacy, and sexual activity. While this relationship has previously been documented among individuals with breast^16^ and testicular cancer,^17^ it has to our knowledge not been studied within the brain tumor population. Notably, Leonetti et al^24^ reported that 27% of 148 patients with low- and high-grade glioma retrospectively felt less sexually attractive after diagnosis compared to before diagnosis, at a mean of 4 years postdiagnosis. Thus, our finding that body image disturbance is associated with sexual dysfunction in young adults with brain tumors, complement and extend previous observations. In a clinical context, validating patients’ experiences and supporting communication with partners may be helpful as psychosocial and relational factors play an important role in follow-up care.^47^ Taken together, the associations between sexual function and depression symptoms and body image disturbance indicate that psychological factors may have a sustained influence on sexual functioning in this population.

Corticosteroid treatment can be used to reduce cerebral edema in patients with brain tumors.^48^ In addition to physical side effects such as weight gain, which can contribute to body image disturbance^46^ corticoids are well known to cause psychological side effects including mood swings, anxiety, and depression.^49^ Such psychological side effects may indirectly interfere with intimacy and sexual functioning. Thus, these mechanisms could partly explain the observed association between body image disturbance and sexual function. As corticosteroid use is not recorded in the National Quality Registry, we were unable to determine which participants received such treatment. However, corticosteroids are typically administered symptomatically in cases of tumor-related edema or increased intracranial pressure^50^; therefore, some participants with malignant tumors were likely to have received this therapy.

Our finding that a substantial proportion reported low interest in sexual activity highlights the importance of addressing intimacy and sex life in clinical care. This is particularly important given that previous studies have shown that health-care professionals often fail to address potential impact on sexual function with individuals with brain tumors.^18^^,^^22^ Considering that these patients have survival rates exceeding 5 years, and remain sexually active, sexual health represents a relevant long-term concern for many who will live long after diagnosis. In this context, it is encouraging that, consistent with Leonetti et al,^24^ erectile function in men and vaginal lubrication in women appeared largely unaffected. Young men concerned about treatment-related erectile dysfunction may be reassured that posttreatment problems are uncommon. Sexual concerns are multifaceted and warrant a holistic approach, and management of sexual problems may involve physicians, nurses, psychologists, or sexologists, depending on the nature of the problem.

Intimate relationships are central in young adulthood. Prior research report a notable decline in partner status from pre- to postdiagnosis,^24^ suggesting that brain tumors may disrupt relationships. Encouragingly, partnership prevalence remained relatively high throughout the 1.5- to5-year follow-up period, and levels of sexual activity were consistently high across assessments. Further, we found that having a partner was associated with greater satisfaction with sex life, indicating a potential protective factor for sexual function. However, our data did not assess relationship quality. Further research is needed to explore the quality of intimate relationships in this population of both patients and their partners.

### Strengths and Limitations

A major strength of this study is the use of a population-based sample identified through the Swedish Quality Registry for CNS tumors (coverage >90%), which enabled access to clinical data and comparisons between responders and nonresponders. The longitudinal design and the use of standardized instruments add robustness to the findings. However, several limitations should be acknowledged. First, the relatively small sample size increases the risk of type II errors, meaning that true associations, such as differences by tumor type or changes over time, may have gone undetected. Second, although dropout and attrition analyses indicated no significant differences between responders and nonresponders, the exclusion of 5 potential individuals, excluded due to their participation in a randomized controlled trial targeting sexual problems (where sexual dysfunction was an inclusion criteria)^51^ may have led to an underestimation of prevalence of sexual dysfunction. The initial response rate was moderate (58%), which could introduce selection bias. Third, we lacked data on corticosteroid treatment and localization of the tumor, both of which may be related to sexual dysfunction.^21^^,^^50^ As this information was not available, we could not examine their potential impact on sexual function, which represents a limitation of the study. Future studies should aim for larger samples and consider mixed methods approaches to capture subjective experiences alongside quantitative methods.

## Conclusion

Most young adults with brain tumors report being sexually active and favorable sexual functioning up to 5 years after diagnosis. A small subgroup reported low interest in sexual activities, underscoring the need to include discussions about possible cancer-related impact on sex life into routine follow-up care.

## Supplementary Material

npag048_Supplementary_Data

## Data Availability

The data that support the findings of this study are available on request from the corresponding author. The data are not publicly available due to privacy or ethical restrictions.
