# Supplementary material for: Sexual function and activity in young adults with a primary brain tumor—A population-based longitudinal study
Source: Neurooncol Pract. 2026 May 22;13(5):973–83. doi: 10.1093/nop/npag048 (PMC13578483; doi:10.1093/nop/npag048)
Supplement: Supplementary file 1 [file npag048_supplementary_data.zip › Supplementary Table 2_260404.docx]

**Supplementary Table 2.** Sociodemographic and clinical characteristics of study participants (n=72) completing all surveys compared to initial non-responders

(n=89) and partial responders who completed one or two surveys (n=42)

|  | | | Study participants  (n=72) | | Initial non-responders  (n=89) | | P-value^a^ | Partial responders (1-2 time points)  n=42 | P-value^b^ |  |
| --- | --- | --- | --- | --- | --- | --- | --- | --- | --- | --- |
|  | | | n (%) | | n (%) | |  | n (%) |  |  |
| Age at diagnosis, Mean (SD) | | | 31.8 (6.2) | | 30.6 (5.9) | | 0.93 | 33.0 (5.6) | 0.31 |  |
| Gender | | |  | |  | | 0.13 |  | 0.15 |  |
| Men | | | 31 (43) | | 49 (55) | |  | 24 (57) |  |  |
| Women | | | 41 (57) | | 40 (45) | |  | 18 (43) |  |  |
| Country of birth | | |  | |  | |  |  | 0.41 |  |
| Sweden | | | 61 (85) | | NA | |  | 33 (79) |  |  |
| Outside Sweden | | | 11 (15) | | NA | |  | 9 (21) |  |  |
| Highest Education | | |  | |  | |  |  | 0.32 |  |
| University degree | | | 40 (56) | | NA | |  | 18 (43) |  |  |
| No university degree^c^ | | | 32 (44) | | NA | |  | 24 (57) |  |  |
| Main occupation | | |  | |  | |  |  | 0.83 |  |
| Working / studying | | | 46 (64) | | NA | |  | 26 (62) |  |  |
| Unemployed, sick-leave, other^d^ | | | 26 (36) | | NA | |  | 16 (38) |  |  |
| Relationship status | | |  | |  | |  |  | 0.51 |  |
| Partnered | | | 57 (79) | | NA | |  | 31 (74) |  |  |
| Not partnered | | | 15 (21) | | NA | |  | 11 (26) |  |  |
| Have children | | |  | |  | |  |  | 0.38 |  |
| Yes | | | 35 (49) | | NA | |  | 24 (57) |  |  |
| No | | | 37 (51) | | NA | |  | 18 (43) |  |  |
| Sexual orientation | | |  | |  | |  |  | 0.35 |  |
| Heterosexual | | | 69 (97) | | NA | |  | 38 (93) |  |  |
| Non-heterosexual | | | 2 (3) | | NA | |  | 3 (7) |  |  |
| **Clinical characteristics** | | | | | | | | | |  |
| Tumor type | | |  | |  | | 0.64 |  | 0.28 |  |
| Benign | | | 33 (48) | | 35 (42) | |  | 15 (36) |  |  |
| Malignant | | | 36 (52) | | 47 (57) | |  | 27 (64) |  |  |
| Primary treatment^e^ | | |  | |  | |  |  |  |  |
| Surgery | | | 63 (88) | | 77 (88) | | 0.81 | 39 (93) | 0.74 |  |
| Chemotherapy | | | 15 (21) | | 22 (25) | | 0.57 | 13 (31) | 0.24 |  |
| Radiotherapy | | | 15 (21) | | 25 (28) | | 0.29 | 14 (33) | 0.15 |  |
| Treatment intensity (ITR-YA)^f^ | | |  | |  | | 0.42 |  | 0.12 |  |
| Least/moderate | | | 53 (76) | | 68 (70) | |  | 26 (62) |  |  |
| Very/most | | | 17 (24) | | 29 (30) | |  | 16 (38) |  |  |
| Abbreviations: SD, Standard deviation; NA, Not applicable  ^a^P-values comparing study participants to initial non-responders  ^b^P-value comparing study participants to partial responders (1-2 time points)  Differences between groups tested with student’s t-test (age) and Chi-square tests or Fisher’s exact test  ^c^Includes elementary school, upper secondary school, folk high school  ^d^Parental leave, retired  ^e^Sum up to more than 100% as participants may have had more than one treatment.  ^f^Treatment intensity according to the Intensity of Treatment Rating Young Adult (ITR-YA) | | | | | | | | | |  |
|  |  |  |  |  |  |  |  |  |  |  |
|  | | |  |  | |  | | | | |
