# Supplementary material for: Sexual function and activity in young adults with a primary brain tumor—A population-based longitudinal study
Source: Neurooncol Pract. 2026 May 22;13(5):973–83. doi: 10.1093/nop/npag048 (PMC13578483; doi:10.1093/nop/npag048)
Supplement: Supplementary file 1 [file npag048_supplementary_data.zip › Supplementary table 1_260412.docx]

**Supplementary Table 1**. World Health Organization (WHO) Classification of Tumours of the Central Nervous System, revised 4th edition (2016), and ICD‑O‑3 codes for tumors in the study sample (n = 72).

| **Tumor type** | **Number** | **WHO- grade** | **Group** |
| --- | --- | --- | --- |
| Meningioma | | | |
| 95300 – Secretory Meningioma | 24 | I | Meningioma |
| 95391 - Meningioma, atypic | 5 | II | Meningioma |
| Low-grade glioma | | | |
| 93823 – Mixed Glioma | 1 | II | Low malignant |
| 93823 - Oligoastrocytoma | 1 | II | Low malignant |
| 94003 – Astrocytoma (low grade) | 2 | II | Low malignant |
| 94503 - Oligodendroglioma, NOS | 4 | II | Low malignant |
| 95061 - Neurocytoma | 1 | II | Low malignant |
| 94513 - Oligodendroglioma, anaplastic | 1 | III | Low malignant |
| High-grade glioma | | | |
| 94013 - Astrocytoma, anaplastic | 1 | III | High malignant |
| 93803 - Glioma, malignant | 2 | IV | High malignant |
| 94403 - Glioblastom multiforme | 20 | IV | High malignant |
| 9470/3 – Medulloblastoma, NOS | 2 | IV | High malignant |
| 94733 - Primitive neuroectodermal tumor (PNET), NOS | 1 | IV | High malignant |
| Benign/other malignant CNS tumors | | | |
| 95600 - Schwannom | 2 | I | Benign |
| 95223 - Olfactoric neuroblastoma (C72.2) | 1 | IV | High malignant |
| 90643 - Germinoma | 1 | II | Low malignant |
| Missing data: n=3 | | | |
